# Ageing, microbes and health

**DOI:** 10.1111/1751-7915.14477

**Published:** 2024-05-27

**Authors:** Paul W. O'Toole

**Affiliations:** ^1^ School of Microbiology University College Cork Cork Ireland; ^2^ APC Microbiome Ireland University College Cork Cork Ireland

## Abstract

The human gut microbiome is a modifier of the risk for many non‐communicable diseases throughout the lifespan. In ageing, the effect of the microbiome appears to be more pronounced because of the lower physiological reserve. Microbial metabolites and other bioactive products act upon some of the key physiological processes involved in the Hallmarks of Ageing. Dietary interventions that delay age‐related change in the microbiome have also led to delayed onset of ageing‐related health loss, and improved levels of cognitive function, inflammatory status and frailty. Cross‐sectional analysis of thousands of gut microbiome datasets from around the world has identified key taxa that are depleted during accelerated health loss, and other taxa that become more abundant, but these signatures differ in some geographical regions. The key challenges for research in this area are to experimentally prove that particular species or strains directly contribute to health‐related ageing outcomes, and to develop practical ways of retaining or re‐administering them on a population basis. The promotion of a health‐associated gut microbiome in ageing mirrors the challenge of maintaining planetary microbial ecosystems in the face of anthropogenic effects and climate change. Lessons learned from acting at the individual level can inform microbiome‐targeting strategies for achieving Sustainable Development Goals at a global level.

## THE MICROBIOME AS A MODULATOR OF NON‐COMMUNICABLE DISEASE RISK

The development of culture‐independent methods allowed the rigorous identification of the complete repertoire of microbes present in a particular sample, and this technological improvement led to the realization that the human gut microbiome displayed altered composition and function in a wide range of pathophysiologies and syndromes (reviewed in Lynch & Pedersen, 2016). Examples of non‐communicable diseases in which an altered microbiome has been implicated include inflammatory bowel disease (Lavelle & Sokol, 2020), irritable bowel syndrome (Jeffery et al., 2020), type 2 diabetes (Pedersen et al., 2016), obesity (Cani & Van Hul, 2023), liver disease (Adolph et al., 2018), cardiometabolic disease (Ghosh & Valdes, 2023) and colon cancer (Marchesi et al., 2011). Mechanisms by which the microbiome may exert these disease‐promoting effects can be assigned to a number of broad categories that include effects on homeostatic control of metabolic, immune or neurological functions (reviewed in ref. Ghosh et al., 2022a, 2022b). However, as has been pointed out, there is a large gap between the number of studies describing microbiome alterations and the number of studies probing these alterations in clinical intervention trials (de Vos et al., 2022).

A common strategy to prove causality is to transfer the altered microbiome and the human disease phenotype to an animal model, but the proportion of human pathophysiologies that can be replicated in animal models by human microbiome transfer is implausibly high (Walter et al., 2020). Furthermore, there is a high degree of overlap across multiple diseases of the microorganisms that are either depleted (usually commensals) or enriched (so‐called pathobionts) (O'Toole et al., 2023). This has led us to conclude that many reported microbiome–disease associations are consequential rather than causative, and that identification of therapeutic targets requires larger longitudinal studies able to adjust for multiple confounders (O'Toole et al., 2023). Non‐communicable diseases with which microbiome alterations have been linked are generally complex multifactorial diseases, usually involving polygenic risk alleles and environmental modifiers. The microbiome is not necessary and sufficient (independently causal) in the aetiology of the vast majority of these diseases, making it difficult or impossible to fulfil Koch's postulates, which are difficult enough to prove even for pathogens that interact with the microbiome (Vonaesch et al., 2018). All this has implications for developing microbiome‐directed therapies.

Although many microbiome‐directed therapeutics are in late‐stage development or already commercially available, the mechanistic evidence for their mode of action is generally poor or absent, except for the low bar of restoring the basic composition and function of the gut microbiome in subjects with *Clostridioides difficile*‐associated diarrhoea caused by broad‐spectrum antibiotics (Louie et al., 2023; Sims et al., 2023). Microbiome modulation is also emerging as an adjunct to cancer immunotherapy based upon the differences in gut microbiome between responders and non‐responders, and the prospect of rescuing the latter patient category (Routy et al., 2023). Other translational medicine based upon microbiome information includes novel diagnostics, such as the improvement of dietary recommendations to manage postprandial glucose levels by including patient microbiome information (Zeevi et al., 2015), and diagnostics for colon cancer (Flemer et al., 2018).

One of the challenges for establishing links between an altered gut microbiome and human disease is defining what is ‘normal’ (Shanahan et al., 2021). Normality is context dependent because of the reciprocal interactions between personal factors (genetics, birth mode, age, gender, location and or migration), microbe transmission (both vertical and horizontal) and environment and lifestyle (encompassing socio‐economic factors, diet and medications). This adds a layer of complexity of determining disease signatures in the microbiome, especially when comparing data from industrialized and non‐industrialized countries (see below).

## HOW DOES THE MICROBIOME AFFECT HEALTH IN OLDER PEOPLE?

The ageing process in animals involves a complex set of changes that reflect deterioration, loss of function and loss of organ or system control, culminating in death, and these changes can be aligned with the 12 Hallmarks of Ageing (Lopez‐Otin et al., 2023). Many activities of the gut microbiome are either known or suspected of being able to impact upon these Hallmarks (reviewed in Bana & Cabreiro, 2019, Simbirtseva & O'Toole, 2024), and in fact ‘dybiosis’ is listed in the revised Hallmarks but not the original set (López‐Otín et al., 2013), reflecting the consolidation of evidence for microbiome involvement. Given that people experience ageing‐related health loss at different rates, even when other factors like genetics, diet and lifestyle are the same or are statistically adjusted for, the gut microbiome emerged as a plausible environmental modifier of accelerated health loss in ageing (Lynch et al., 2015).

Even in the era of culture‐dependent ‘microflora’ analysis, it was known that older people had a different gut community than their younger counterparts (reviewed by O'Toole & Claesson, 2010). Nearly two decades of research have established that the gut microbiome changes as a function of chronological age and in a way that interacts with health; for comprehensive reviews, see (Bana & Cabreiro, 2019; Ghosh et al., 2022a, 2022b). The details of microbiota taxa and functions that have been reported to differ in older people will not be exhaustively recapitulated here, but rather I will focus on the key factors that govern these differences, and the main take‐away messages. The first message to convey is that age‐related changes in the microbiome are the continuation of a lifelong natural process of succession and replacement beginning at birth (Martino et al., 2022). The period of late childhood through middle age is typically characterized by microbiome stability, unless there is community disturbance due to disease or medication, whereas the microbiome is in flux at the extremes of life (Salazar et al., 2014). There is no single determining adult age at which an ageing‐related gut microbiome appears, but rather a continuous and gradual separation by compositional comparison, and by monitoring microbiome relatedness by beta diversity as a function of age (Falony et al., 2016; Martino et al., 2022; O'Toole & Jeffery, 2015; Xu et al., 2019; Zhang et al., 2021). The gradual change in microbiome composition (Jeffery et al., 2016) and the presence of alternative health‐related microbiome directions (Claesson et al., 2012) suggest that it may be possible to intercept the trajectory of the microbiome at an earlier age, so that intervention becomes an issue of microbiome management/maintenance rather than microbiome restoration.

Some early culture‐independent studies suggested that the microbiome changed with ageing primarily at a macro/phylum level (Claesson et al., 2011; Mariat et al., 2009). However, the description of enterotypes (Arumugam et al., 2011) and their linkage, specifically the Bacteroidetes enterotype, with a low‐fibre, high‐fat and protein diet (Wu et al., 2011), indicated that dietary patterns (recorded in some of those studies Claesson et al., 2012) likely contributed to this phylum‐level disparity. Analysis of larger cohorts revealed a transition from a core microbiome through a reduced core microbiome, and a diversity‐associated module, ultimately to what we termed a ‘long‐stay associated module’, comprising taxa that were more abundant in frail people in long‐term residential care (Jeffery et al., 2016). Other studies reported the loss of organisms recognized in the literature as beneficial, and the gain of organisms associated with inflammation or the production of metabolites associated with disease (Xu et al., 2019). A large number of studies have focussed on the microbiome of centenarians (Biagi et al., 2010; Collino et al., 2013; Luan et al., 2024; Odamaki et al., 2016; Pang et al., 2023; Rampelli et al., 2020; Wu et al., 2019), which represents a special case of extreme ageing. As we have discussed at length in a previous review (Ghosh et al., 2022a, 2022b), reaching extreme age is not necessarily a model for how to stay healthy during ageing, since many centenarians have a significant disease burden, notwithstanding the fact that some studies report youth‐associated gut microbiome signatures in centenarians (Pang et al., 2023).

We have analysed progressively larger datasets to provide inventories of gut microbiome taxa that change in abundance during ageing, and that show correlations with health status across multiple studies (Ghosh, Das, et al., 2020; Ghosh, Rampelli, et al., 2020). As an overarching generalization, biological ageing is marked by the loss of species in the genera *Faecalibacterium, Roseburia, Coprococcus, Bifidobacterium, Prevotella* and *Eubacterium rectale* (Group 1 taxa Ghosh et al., 2022a, 2022b). Group 3 taxa that increase with healthy ageing include species in the genera *Akkermansia, Christensenellaceae, Odoribacter, Butyricimonas, Butyrivibrio, Barnesiella* and *Oscillospira*. Finally, a large and more variable set of taxa in Group 3 are associated with unhealthy ageing, and they include *Eggerthella*, *B. fragilis*, *Clostridium hathewayi, C. bolteae, C. clostridioforme, C. scindens, Ruminococcus torques, R. gnavus, Bilophila, Veillonella* and the *Enterobacteriaceae* (Ghosh et al., 2022a, 2022b). Some of these organisms are among those that show either depletion or enrichment in microbiome‐related diseases (Duvallet et al., 2017; Ghosh, Das, et al., 2020; Ghosh, Rampelli, et al., 2020), which, as noted above, may indicate that these alterations are consequential (O'Toole et al., 2023). However, there are microbiome changes that are specific to unhealthy ageing, and while direct evidence for mechanisms is still lacking, these taxa have been linked to the production of metabolites including deoxycholic and lithocolic acids, para‐cresol, ethanol, acetone, ammonia and trimethylamine (Ghosh, Das, et al., 2020; Ghosh, Rampelli et al., 2020). These metabolites are associated with a variety of diseases (reviewed by us in Ghosh et al., 2022a, 2022b) and can readily be linked to effects upon the Hallmarks of Ageing. The sporadic form of colorectal cancer is an age‐related disease, and the altered microbiome associated with colon cancer (Janney et al., 2020) can be exploited for diagnostic purposes (Thomas et al., 2019).

Summary statistics on the microbiome are attractive for reducing data complexity, and high microbiome diversity is often invoked as a desirable trait because lower microbiome diversity is associated with various diseases (Valdes et al., 2018). In the case of ageing, we noted that passage through a higher‐diversity disturbed microbiome state was a feature prior to the acquisition of disease‐associated taxa (Jeffery et al., 2016) and is thus undesirable. Another recent study reported no association between microbiome alpha diversity and chronological age, biological age or physical capacity in either sex (Tzemah‐Shahar et al., 2024), while analysis of data from the American Gut Project reported that the alpha diversity of gut microbiota increased with increasing age (Zhu et al., 2020). Another summary statistic that has attracted attention is microbiome *uniqueness*, a measure of how different microbiome datasets are from each other. Wilmanski and colleagues reported that retention of a *Bacteroides*‐dominated microbiome and low levels of uniqueness collectively predicted health in a 4‐year follow‐up of survival in elderly subjects (Wilmanski et al., 2021), and that retention of youth‐associated microbiome negatively correlated with survival. Aspects of this were corroborated in a recent study of metabolic diseases in older subjects, whereby subjects with disease have an apparently ‘younger’ microbiome (Fu et al., 2023). When we recently compared a number of summary statistics as appropriate measures of microbiome changes in unhealthy ageing, we found that Kendall uniqueness was the best indicator of loss of the core microbiome and the abundance and ranking of disease‐associated and health‐associated taxa (Ghosh et al., 2022a, 2022b). However, we also need to take account of geographical effects that interact with alpha‐ and beta‐diversity metrics, discussed in the following section.

## MICROBIOMES AND HEALTH IN A CHANGING WORLD

The gut microbiome present in an individual at any given time is largely a reflection of their exposure to a range of environmental factors, current and previous (Falony et al., 2016; Gacesa et al., 2022; Rothschild et al., 2018; Vujkovic‐Cvijin et al., 2020). These factors contribute to the problem that human microbiome variance is underestimated (Shanahan et al., 2023). Overlaid on this is the fact that, despite recent efforts to address it, surveys of the human gut microbiome are dominated by those of citizens of developed countries and powerful economies (Abdill et al., 2022). Another relevant feature is the fact that aboriginal and non‐industrialized societies harbour gut microbiomes that are usually of high diversity and complexity, and that these features are lost when these countries become industrialized (Shanahan et al., 2022). Examples of this phenomenon include the >90,000 microbial genomes identified by ultradeep sequencing in Hadza hunger gatherers and that are absent in western datasets/individuals (Carter et al., 2023); the presence of taxa in Irish Travellers shared with indigenous people around the world, and which are being lost on integration into the settled community (Keohane et al., 2020); and the extraordinarily diverse and function‐rich microbiome of uncontacted Amerindian people (Clemente et al., 2015).

The intimate connection between lifestyle and microbiome ecology is also highlighted by the findings of a comparison between subjects in Papua New Guinea and the United States, which concluded that bacterial dispersal was the main ecological force governing the relatedness of the microbiome across subjects in Papua New Guinea, whereas selection linked to relatively higher cultural, dietary and genetic heterogeneity was the dominant ecological force shaping the US gut microbiome (Martinez et al., 2015). The same authors achieved similar conclusions reanalysing published microbiome data of Italian and Hadza hunter gatherers. These observations raise profound questions such as: is the microbiome of people living in an industrialized world incompatible with human biology? (Sonnenburg & Sonnenburg, 2019); should we catalogue and biobank vanishing gut microbes for future use as biotherapeutics for non‐communicable microbiome‐associated diseases? (Bowers, 2023); what are the health consequences for ethnic minorities harbouring ancestral microbiomes that are forcibly integrated into a larger community, or forcibly relocated because of geopolitical events? (Shanahan et al., 2022).

Integrating what was described above in this review about how microbiomes are a risk factor for non‐communicable disease, that they are particularly important in older subjects that are losing physiological resilience anyway, and that they are governed by factors such as socialization and habitual diet, it becomes obvious that so‐called western civilization has created very challenging conditions for healthy ageing. We believe this is reflected in our observation that there are robust associations between age and microbiome revealed by beta‐diversity analysis, and between microbiome alpha diversity and uniqueness, in Europe and North America, but not in Africa and South East Asia (Ghosh et al., 2022a, 2022b). One interpretation of this is that a non‐industrialized lifestyle reduces the rate of ‘deterioration’ of the gut microbiome with age, which means retention of commensals and delayed outgrowth of pathobionts, whereas a western lifestyle accelerates this deterioration. Although modern medicine in industrialized societies offers unparalleled opportunities to treat major diseases, in order to extend health‐span, not just lifespan, medicine would arguably benefit from a broader society‐based holistic approach, embracing all factors that impact on healthy ageing including the microbiome. The final section explains how embracing sustainability should be part of this approach.

## GUT MICROBIOME MANAGEMENT IS EMBLEMATIC OF THE GLOBAL ECOLOGICAL CHALLENGE

Acclaimed microbiologist, the late Stanley Falkow is often quoted as having told his students that ‘the whole world is covered in a fine patina of feces’; it is no exaggeration to point out that we share some of our gut microbe communities with each other and with the global environment, and that microbiome science is merely a reinvention and niche application of environmental microbiology. As we begin to consider the necessity of managing our internal gut microbiomes to reduce the risk of non‐communicable disease and to promote healthy ageing, it is against the backdrop of the unprecedented challenge of climate change caused by human activity. The practical aspects of that challenge are increasingly obvious, even to the most persistent deniers in society, but we still lack the collective political and economic will to make the necessary interventions to effectively tackle global warming and climate change. Microbiologists have always been aware of the important role that microbes play in the critical biogeochemical cycles that are effected by climate change (Sokol et al., 2022), particularly the production and consumption of greenhouse gases, and have clearly articulated the need to review and manage microbial ecosystems as part of implementing the Sustainable Development Goals (SDGs) (Cavicchioli et al., 2019). In a recent status review, Janet Jansson highlighted the potential roles of microbes in sequestering carbon dioxide and methane, for biodegradation of pollutants, and for allowing plants to grow under climate stress (Jansson, 2023). I believe that the principles of the SDGs that apply to the improvement of the global microbial environment resonate with the strategies necessary to improve the human gut microbiome (O'Toole & Paoli, 2023), and in fact, there are sensible, mutually beneficial ways to link the two aims. The most obvious example is the desirability of shifting the human diet towards sustainably produced, minimally processed foods, which would benefit consumer and global environment alike. The fact that switching to a Mediterranean diet promotes healthy ageing (Ghosh, Das, et al., 2020; Ghosh, Rampelli, et al., 2020) illustrates the benefit of consuming locally produced, minimally processed foods that are more plant‐based and thus contribute less to greenhouse gas emissions. The sensitivity of global food supply chains to the early days of the COVID‐19 lockdowns, based on just‐in‐time shipping and block‐chain inventory management, highlights the degree to which consumers in industrialized countries have become totally dependent on long‐range food supplies, usually with a significant proportion of highly processed items.

With regard to *SDG3 Ensure healthy lives and promote well‐being for all at all ages*, the prospect of microbiome therapy to rectify the internal human microbiome (Ratiner et al., 2023; Sorbara & Pamer, 2022) resonates with the ambition to use microbes in climate change mitigation strategies (Jansson, 2023). Individuals tend to become more exercised about managing their health as they grow older, and the current upswell in consumer interest in the microbiome offers the prospect of promoting basic microbiology knowledge across society, supporting *SDG4 Quality Education*, which could in turn lead to greater interest in global sustainability. The analysis presented in this review on differences in the microbiome of industrialized and non‐industrialized countries, and on the putative health ramifications of enforced movement or increasing westernization, highlights the need for greater understanding of the gut microbiome and health in the Global South and least developed countries (O'Toole & Paoli, 2023), linking logically with *SDG 10: Reduced Inequalities* and *SDG 11: Sustainable Cities and Communities*, among other *SDG*s.

In conclusion, there is a logical resonance between managing microbial ecosystems on an individual basis and on a planetary basis, and both endeavours may contribute to achieving Sustainable Development Goals. We need to act quickly on a planetary basis to make individual interventions worthwhile.

## AUTHOR CONTRIBUTIONS

**Paul W. O'Toole:** Writing – original draft; writing – review and editing; conceptualization.

## FUNDING INFORMATION

Work in PWOT's laboratory is supported by Science Foundation Ireland through a Centre Award to APC Microbiome Ireland (12/RC/2273_P2), and by the European Union's Horizon Europe research and innovation programme under grant agreement no. 101079777 with the MicrobAIome consortium, and agreement no. 101083671 with the Microbiomes4Soy consortium.

## CONFLICT OF INTEREST STATEMENT

None.
